# Cytotoxic Activity and In Silico Study of Secondary Metabolites Derived from *Dactylospongia elegans*

**DOI:** 10.3390/md24080271

**Published:** 2026-08-04

**Authors:** Yuni Elsa Hadisaputri, Nafisa Nurfatia Hidayat, Tutik Murniasih, Ariyono Hadi, Mutakin Mutakin, Nunung Yuniati, Yonathan Asikin, Elin Julianti

**Affiliations:** 1 Department of Pharmaceutical Biology, Faculty of Pharmacy, Universitas Padjadjaran, Jatinangor 45363, Indonesia; nafisa21001@mail.unpad.ac.id; 2 Graduate School, Universitas Padjadjaran, Bandung 40132, Indonesia; 3 Research Center for Vaccine and Drugs, Research Organization for Health, National Research and Innovation Agency (BRIN), Bogor 16911, Indonesia; tutikmurniasih71@gmail.com (T.M.); ari_080885@yahoo.com (A.H.); 4 Department of Pharmaceutical Analysis and Medicinal Chemistry, Faculty of Pharmacy, Universitas Padjadjaran, Jatinangor 45363, Indonesia; mutakin@unpad.ac.id; 5 Laboratory of Pharmacology and Toxicology, Department of Pharmacology and Clinical Pharmacy, Faculty of Pharmacy, Universitas Gadjah Mada, Yogyakarta 55281, Indonesia; nunung@mail.ugm.ac.id; 6 Department of Bioscience and Biotechnology, Faculty of Agriculture, University of the Ryukyus, Nishihara 903-0213, Japan; y-asikin@cs.u-ryukyu.ac.jp; 7 The United Graduate School of Agricultural Sciences, Kagoshima University, Kagoshima 890-0065, Japan; 8 Department of Pharmacochemistry, School of Pharmacy, Institut Teknologi Bandung, Bandung 40132, Indonesia

**Keywords:** *Dactylospongia elegans*, MDA-MB-231, cytotoxicity, 5-epi-illimaquinone, calciferol, breast cancer

## Abstract

Breast cancer remains a major global health burden. *Dactylospongia* species have been explored for their cytotoxic potential. This study aims to evaluate the cytotoxic potential of compounds derived from the marine sponge *Dactylospongia elegans*. *Dactylospongia elegans* were collected from the Lembeh Strait, macerated using methanol, then partitioned to an ethyl acetate fraction. The cytotoxic activity of these fractions was assessed using MDA-MB-231 cells while toxicity testing was done using the BSLT. TLC was carried out to determine the groups of compounds, while LC-MS/MS was used to predict active compounds contained in the ethyl acetate fractions. In silico studies were conducted as preliminary studies to determine the antitumor mechanism. The ethyl acetate fraction and F4 subfraction of *Dactylospongia elegans* exhibited cytotoxicity toward MDA-MB-231 cells with IC_50_ values of 15.72 and 41.76 µg/mL, respectively. The BSLT indicated the strongest toxicity belongs to the F6 subfraction (LC_50_ = 32.831 µg/mL). TLC analysis confirmed the presence of major secondary metabolites as terpenoids, steroids, and alkaloids, then confirmed with LC-MS/MS including 5-epi-illimaquinone and calciferol. Molecular docking revealed that calciferol exhibited the strongest binding affinity toward tyrosine kinase and p53–MDM2 receptors, with binding energies of −10.13 and −10.28 kcal/mol, respectively. These findings suggest that *Dactylospongia elegans* contains bioactive constituents with potential anticancer activity, particularly against TNBC.

## 1. Introduction

Cancer refers to a group of diseases characterized by uncontrolled cell division and the ability to invade other tissues [1]. In 2022, there were approximately 20 million new cancer cases and 9.7 million related deaths worldwide [2]. Breast cancer was the most commonly diagnosed cancer in females, with an estimated 2.3 million new cases. Indonesia has a high breast cancer burden, recording 66,271 new cases and over 22,000 deaths, accounting for a significant portion of the country’s total cancer cases [3].

Current breast cancer treatments, including surgery and chemotherapy, have limitations. Surgery is typically used for early-stage cases without metastasis, while chemotherapy can be applied at any stage [4]. Chemotherapy drugs are potent but lack specificity, also resistance in chemotherapy drugs is one problem in breast cancer therapy nowadays [5].

Of the breast cancer categories in four types of cells, triple-negative breast cancer (TNBC) is the most difficult to treat. MDA-MB-231 cells are a highly invasive TNBC cell line that lack estrogen, progesterone, and HER2 receptors, making them resistant to hormonal and targeted therapies [6]. Due to the aggressive nature of TNBC, alternative treatment strategies are urgently needed. Research has increasingly focused on discovering more selective and safer anticancer agents, including those derived from natural marine resources.

Marine sponges are among the simplest and oldest known animals, with a fossil record dating back over 580 million years. They anchor to hard ocean substrates and filter-feed on bacteria and microscopic particles, contributing to water purification. Sponges inhabit diverse environments worldwide, from freshwater to deep-sea ecosystems, playing a crucial role in the benthic zone [7]. While their true diversity may be higher, approximately 15,000 species have been identified, mainly within the *Demospongiae*, *Hexactinellida*, *Homoscleromorpha*, *Hymedesmiidae*, and *Calcarea* families [8].

Sponges of the genus *Dactylospongia* (family Dictyodendridae) are abundant in tropical and subtropical marine regions, including Indonesia and Polynesia, and are recognized for producing diverse bioactive metabolites, primarily sesquiterpenes, sesterterpenes, sterols, and pregnanes [9]. Despite limited research on *Dactylospongia elegans* as a cytotoxic agent, studies have identified illimaquinone and smenospongine from *Dactylospongia elegans* as potent against BACE1 (Mesenchymal cell lines) with IC_50_ values of 65 µM and 78 µM, respectively. Against U251MG (human glioblastoma multiforme), smenospongine showed cytotoxic activity with an IC_50_ value 2.4 µM. Additionally, smenospongidine had an IC_50_ value of 12.6 µM against Panc-1 (Pancreas cancer) [10]. In another study, 5-epi-illimaquinone and pelorol also reported cytotoxic activity against 501-mel melanoma cells with IC_50_ values of 7.88 µg/mL and 12.51 µg/mL, respectively [11]. Previous studies suggest *Dactylospongia elegans* has promising cytotoxic activity, particularly against brain, pancreas [10] and melanoma cancer cells [11]. However, this study aimed to evaluate the cytotoxic activity of the ethyl acetate fraction of *Dactylospongia elegans* against MDA-MB-231 cells, determine the toxicity of its fractions using the Brine Shrimp Lethality Test (BSLT), and predict the molecular interactions of identified metabolites through a docking analysis. The findings are expected to contribute to the discovery of new anticancer agents from marine natural products.

## 2. Results

### 2.1. Characterization of Sponge

**Figure 1:**
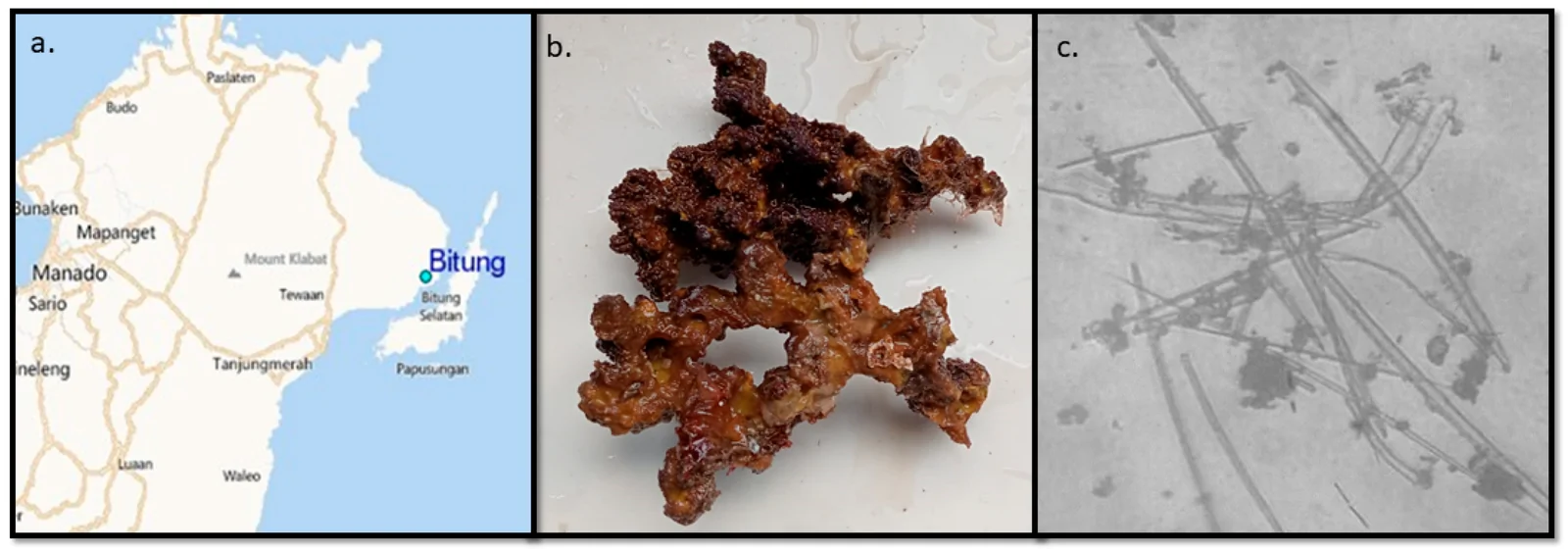
Sponge sample information. (**a**) The map of samples collected, dive point Tanjung Kusu Kusu, Lembeh Strait, Bitung City, North Sulawesi Province, Indonesia. (**b**) Photo of a *Dactylospongia elegans* sponge just after it was taken from the sea. (**c**) *Dactylospongia elegans* sponge spicules for morphological genus identification.

The sponge exhibits an irregularly lobed morphology with a predominantly dark brown coloration and a slightly glossy surface when wet (Figure 1b). The outer texture is uneven and nodular, with a compact yet soft consistency when pressed. Oscula are not distinctly visible on the surface. The internal structure shows a fibrous arrangement typical of the order *Dictyoceratida*. Microscopic observation reveals the presence of spongin fibers with embedded spicules (Figure 1c). The spicules observed are megascleres in a smooth form, which are diactine. No microscleres were detected. Based on the taxonomy handbook of sponges, the sponge is identified as belonging to the genus *Dactylospongia* [12].

### 2.2. Identification Sponge by DNA Sequencing

To complement morphological evidence, DNA barcoding was performed. The sequence obtained from sample G-4282-1 (763 bp) showed >95% similarity to sponge sequences in NCBI BLAST (https://blast.ncbi.nlm.nih.gov/Blast.cgi?PROGRAM=blastn&BLAST_SPEC=GeoBlast&PAGE_TYPE=BlastSearch accessed on 22 March 2026) and clustered within the same clade as *Dactylospongia elegans* (GenBank: KY970158.1, voucher number WAMZ69706, and NCI066).

The sponge partial DNA detected by the sequencer:

Sequence assembly 763 bp;

1 AAACTAACAA GGATTCCCTC AGTAACGGCG AGCGAAGTGG GAATAGCTCA AATTTGAAAT
61 CTCCAGTGCC TGCACTGGCG AATTGTAGTC TGTAGGGCCA GTTTCGCCCG AGATGGTGTC
121 CCCAAAGTCG TCCTGGAAAG GCGGGCCAGA GAGGGTGAGA GCCCCGTGCG TGGGGGCACC
181 GTCGAAGGTA AGATCTGTGC CCAAAGAGTC GGGTTGTTTG GGATTGCAGC CCAAAATGGG
241 TGGTAAACTC CATCTAAAGC TAAATATTGG CACGAGACCG ATAGCGAACA AGTACCGTGA
301 GGGAAAGATG AAAAGCACTT TGAAAAGAGA GTCAAACAGT ACGTGAAACC GTCAGGAGGG
361 AAACGAACGA GGTCGGTGCA GTGATCGCGC AGGTTCAAGC CCTGCCGAAG GCGCCGACGC
421 GACGACATAG CTTGTCCCGT GTCGTACGCC GGAGGCAGTG CCGTACTCCT CGTGAGCACG
481 GACCAGCAGC GGTTGAGCCG GAGCCAGAAG ACCGCAGGGA AGGTGAGTCG ATGAGCACCT
541 GTCGCACATT GACGGCATAT AGCCCTGAGG CACGGCGCTC CGGTCCGACC GAGGAAGGAG
601 GGCAGCCCTT GCCCGTCGTG GTACACGCCG TATGGCGGGG CCTCCCGACA CAAGTGAACC
661 ATGCGGTGTG GACTGCATGC AGTGCACATC GTGGTGGGGA TCTTGTGGCG GAGAGTGCCA
721 CCTGTGTGCC AGTGCTGCTG GTATTCAGAC AGCCTCGTTC GAC

The sequence result got a hit in the NCBI database and was identified as the sponge *Dactylospongia elegans*.

### 2.3. Extraction and Fractionation of Dactylospongia elegans

The sponge *Dactylospongia elegans* was macerated in methanol to collect polar and non-polar compounds. The macerate was then concentrated with a rotary evaporator at 37 °C and a crude extract weight of 15.11 g was obtained. The liquid partition was carried out using ethyl acetate and water (1:1, *v*/*v*) to obtained semipolar compound. The results of the partition were concentrated again and obtained the weight of the ethyl acetate fraction of 0.3113 g (2.06% yield) and the water residue of 5.0128 g (33.17% yield). The ethyl acetate fraction obtained was then identified for its secondary metabolites and evaluated for its cytotoxic activity against MDA-MB-231 cells.

The ethyl acetate fraction of *Dactylospongia elegans* was then further fractionated via normal-phase column chromatography using a gradient elution system of *n*-hexane–ethyl acetate (10:0 to 0:10, *v*/*v*), followed by methanol as the final washing step. The column chromatography separated the ethyl acetate fraction into 10 subfractions.

### 2.4. Secondary Metabolites in Dactylospongia elegans Fraction

The identification of compound groups contained in the ethyl acetate sponge *Dactylospongia elegans* fraction was carried out by a thin layer chromatography (TLC) method. Sulfuric acid (H_2_SO_4_) was used as a universal visualization reagent to detect organic compounds. Furthermore, specific detection reagents confirmed the presence of alkaloids (Dragendorff’s reagent), steroids (Liebermann–Burchard reagent), as well as steroids and terpenoids (*p*-anisaldehyde reagent). In contrast, phenolics were not detected, as indicated by a negative result with FeCl_3_ (Supplementary Figure S1).

### 2.5. LC-MS/MS Analysis

The LC–MS analysis of the ethyl acetate fraction of *Dactylospongia elegans* revealed the presence of several bioactive metabolites with distinct retention times and accurate mass measurements (Table 1).

**Figure 2:**
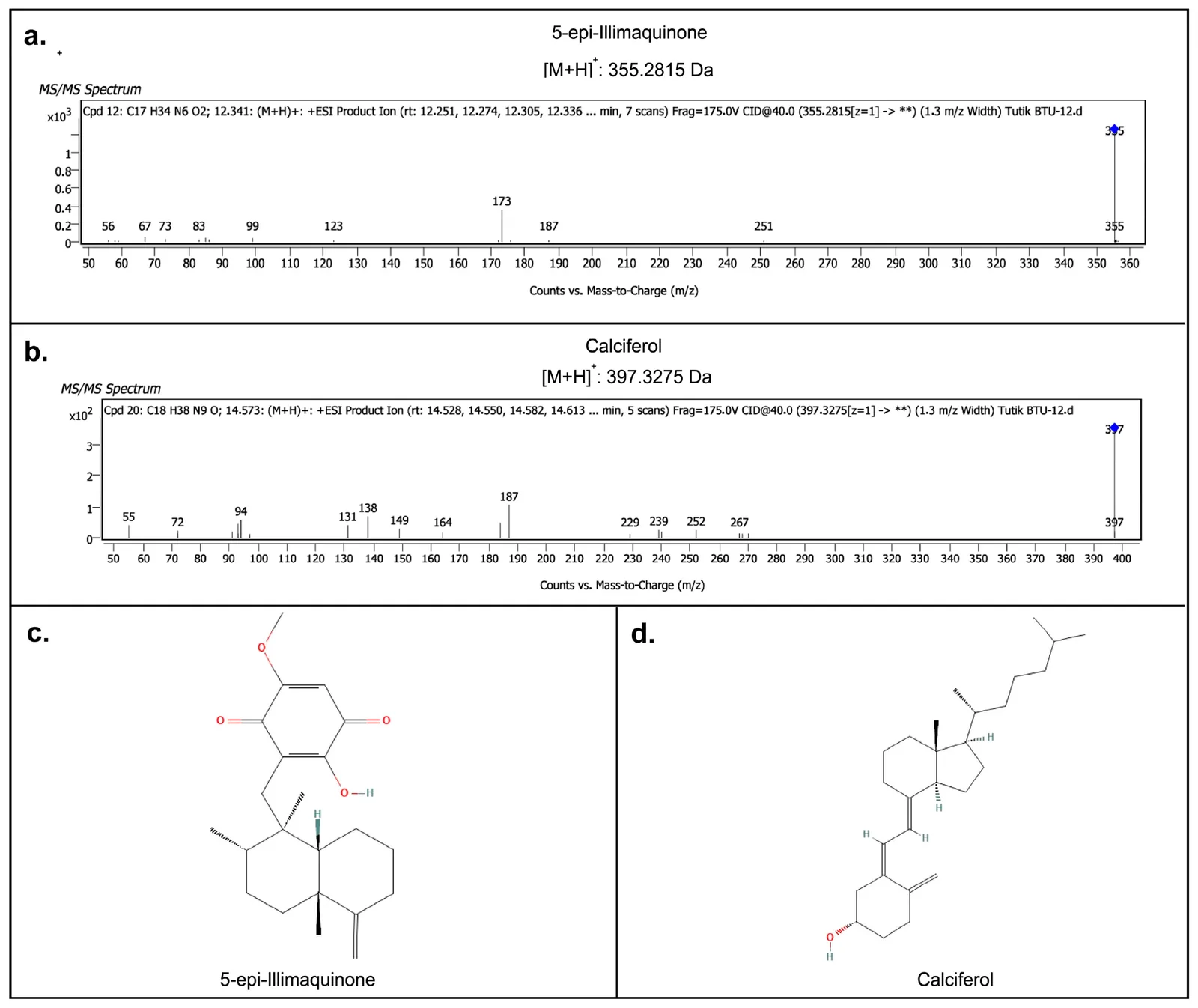
LC-MS/MS results of the secondary metabolites from the ethyl acetate fraction of *Dactylospongia elegans* suspected to be responsible for cytotoxic activity. (**a**) MS spectrum of 5-epi-illimaquinone and (**b**) MS spectrum of Calciferol. (**c**) Structure of predicted compound 5-epi-illimaquinone and (**d**) structure of predicted compound Calciferol.

At a retention time of 9.559 min, a compound with a measured mass of 456.2125 [M + H]+ was identified as Communesin A, previously reported from marine algae *Enteromorpha intestinalis*. Another peak was detected at 11.692 min, corresponding to Andrastine D (*m*/*z* 428.3112), a metabolite commonly derived from marine fungi such as *Penicillium sp.* Additionally, the chromatogram indicated the presence of Cortisol (*m*/*z* 362.2028) at 11.741 min and Myricetin (*m*/*z* 319.2848) at 11.776 min, both of which have been previously reported from marine sources. A notable peak at 12.341 min showed a mass of 354.2745 [M + H]+, predicted as 5-epi-illimaquinone, a well-documented metabolite from *Dactylospongia elegans* (Figure 2a) [11]. Finally, a compound detected at 14.573 min with *m*/*z* 396.3203 was identified as Calciferol, a marine algae-derived metabolite (Figure 2b). The structure and MS spectrum of Communesin A, Andrastine D, Myricetin and Cortisol can be seen in Supplementary Figure S2.

### 2.6. Toxicity Using Brine Shrimp Lethality Test (BSLT)

The toxicity profiles of subfractions from *Dactylospongia elegans* were evaluated using BSLT. The LC_50_ values for all fractions are presented in Table 2. Subfractions F3, F4, F5, F6, and F7 demonstrated high toxicity, with LC_50_ values ranging from 32.831 µg/mL to 67.302 µg/mL. Moderate toxicity was observed in subfractions F2 and F9, with LC_50_ values of 307.407 and 195.396 µg/mL, respectively. Subfraction F1 exhibited low toxicity with an LC_50_ of 685.118 µg/mL. Meanwhile, subfractions F8 and F10 showed no toxic activity, with LC_50_ values exceeding 1000 µg/mL.

### 2.7. Cytotoxic Activity of Ethyl Acetate Fraction and F4 Subfraction Against MDA-MB-231 Cells

The cytotoxic effects of the ethyl acetate fraction and F4 subfraction of *Dactylospongia elegans* were evaluated on the MDA-MB-231 breast cancer cell line and compared with cisplatin as a reference drug.

**Figure 3:**
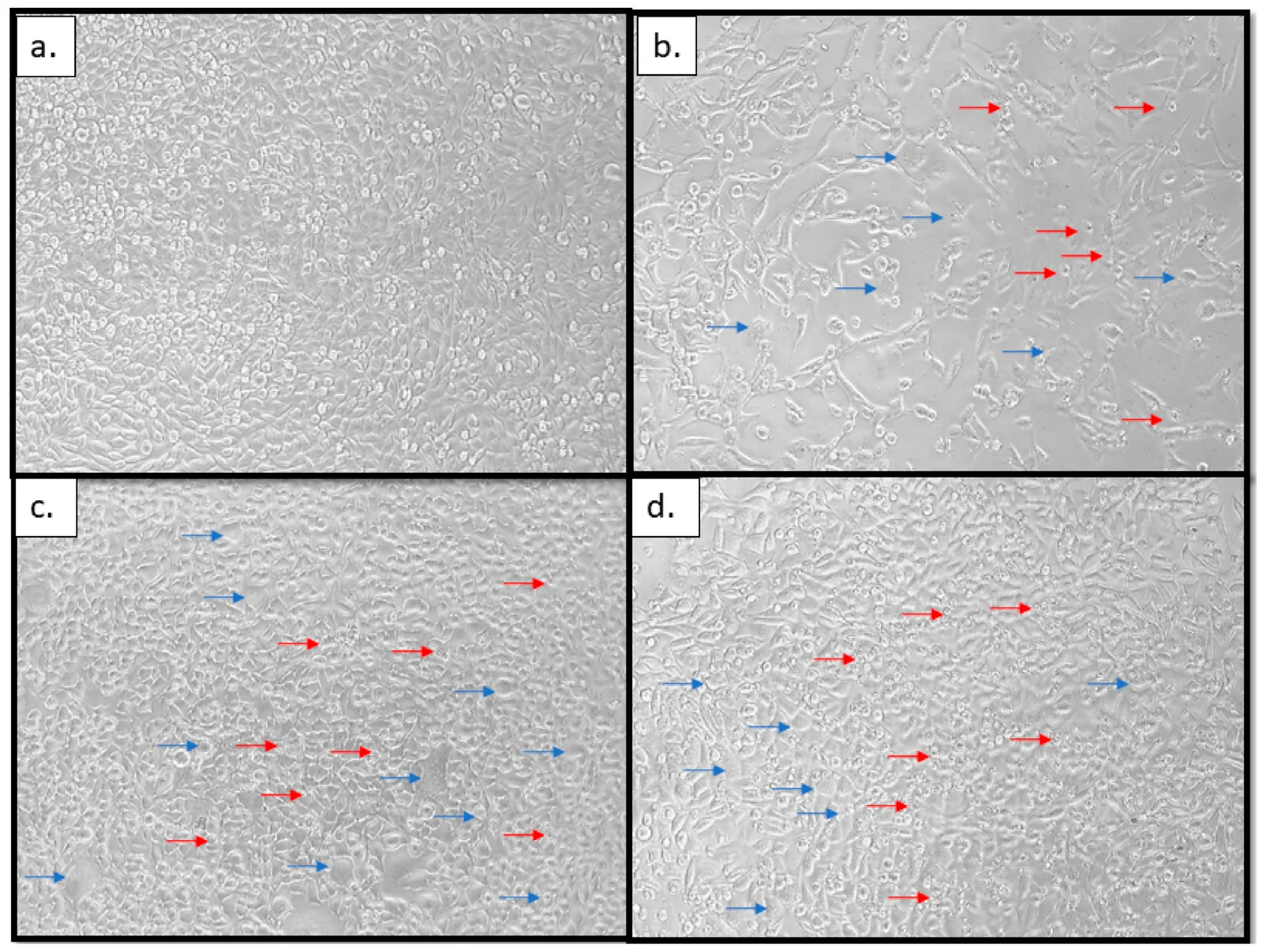
Photograph of MDA-MB-231 cells: (**a**) untreated, (**b**) treated with *Dactylospongia elegans* ethyl acetate fraction, (**c**) *Dactylospongia elegans* F4 subfraction, and (**d**) cisplatin, each at a concentration of 15.625 µg/mL. The blue arrows point to morphological changes signs, including blebbing of cells, and the red arrows point to the cell shrinkage. Both blebbing of cells and cell shrinkage are signs of programmed cell death or apoptosis. Magnification 50×.

As shown in Table 3 and Figure 3, the IC_50_ value of *Dactylospongia elegans* ethyl acetate fraction was 15.72 ± 3 µg/mL, whereas cisplatin showed almost twice the IC_50_ value of 30.99 ± 3 µg/mL, indicating the ethyl acetate fraction of *Dactylospongia elegans* showed stronger cytotoxic activity. Unfortunately, the IC_50_ value of the F4 subfraction of *Dactylospongia elegans* was less cytotoxic than the ethyl acetate fraction at 41.76 ± 0.5 µg/mL, categorizing it as moderate.

Morphological observations under a light microscope (Figure 3) showed clear differences before and after treatment with the *Dactylospongia elegans* ethyl acetate fraction and F4 subfraction, and when treated with cisplatin as a positive control. Untreated MDA-MB-231 cells (Figure 3a) appeared confluent, with a spindle-like shape and intact cell membranes. After treatment with 15.625 µg/mL of the ethyl acetate fraction of *Dactylospongia elegans* (Figure 3b), the F4 subfraction of *Dactylospongia elegans* (Figure 3c) and cisplatin (Figure 3d), the cells showed morphological changes such as blebbing (blue arrow), rounding, detachment, and membrane shrinkage (red arrows), suggesting cytotoxic effects consistent with cell death. Moreover, in MDA-MB-231 cells treated with the ethyl acetate fraction of *Dactylospongia elegans* (Figure 3b), the numbers seemed fewer than in those treated with cisplatin (Figure 3d) or with F4 subfraction of *Dactylospongia elegans* (Figure 3c). his phenomenon indicated that besides apoptosis, cell cycle arrest also occurred after cells were treated with the ethyl acetate fraction of *Dactylospongia elegans*.

### 2.8. Molecular Docking of Predicted Active Compounds Contained in Ethyl Acetate Fraction of Dactylospongia elegans

Molecular docking simulations were performed to evaluate the binding affinities and interaction profiles of six selected compounds, identified through LC–MS analysis of *Dactylospongia elegans* against two cancer-related protein targets: the tyrosine kinase receptor (PDB ID: 1T46) and the p53–Murine Double Minute 2 (MDM2) complex (PDB ID: 4OQ3).

Table 4 shows the validation of molecular docking using native ligands. Validation of the docking protocol was performed using the native ligands of the respective target proteins.

In the case of tyrosine kinase (PDB ID: 1T46), its natural ligand, 4-(4-methylpiperazin-1-ylmethyl)-N-[4-methyl-3-(4-pyridin-3-yl-pyrimidin-2-ylamino)phenyl]benzamide, showed a binding affinity of −13.53 kcal/mol with a root mean square deviation (RMSD) of 0.88 Å and an inhibition constant (Ki) of 120.18 pM. Meanwhile, the native ligand of the p53-MDM2 complex (PDB ID: 4OQ3), identified as 1-(5-chloro-2-methylphenyl)-5-(3-chlorophenyl)-2-(3-methylphenyl)-1H-imidazole-4-carboxylic acid, produced a binding energy of −10.96 kcal/mol, with an RMSD value of 0.49 Å and a Ki of 9.22 nM.

**Figure 4:**
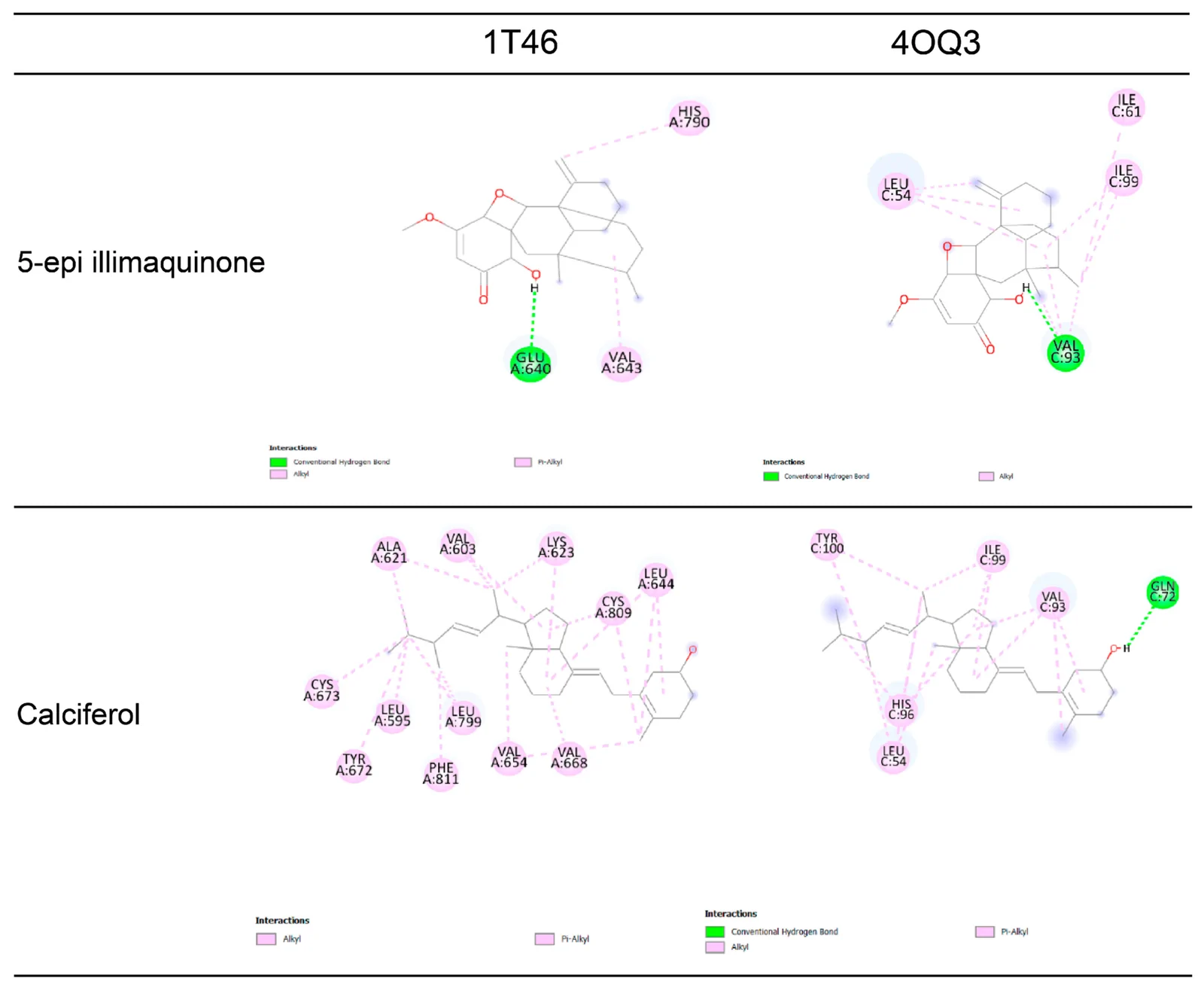
Ligand–receptor interactions of *Dactylospongia elegans* secondary metabolite compounds 5-epi-illimaquinone and Calciferol against 1T46 and 4OQ3 receptors.

The docking outcomes are presented in Table 5, with representative ligand–receptor interaction diagrams shown in Figure 4.

Among the six tested ligands, 5-epi-ilimaquinone showed favorable binding to both targets, with a binding energy of −6.25 kcal/mol and an inhibition constant of 26.07 µM for 1T46, stabilized by one hydrogen bond (GLU640) and two hydrophobic contacts (HIS790, VAL643). Against 4OQ3, it exhibited a stronger affinity (−7.53 kcal/mol; 2.9 µM), forming one hydrogen bond (VAL93) and three alkyl interactions (ILE99, ILE61, LEU54). Andrastin D displayed moderate binding to 1T46 (−5.33 kcal/mol; 67.88 µM), stabilized by one alkyl interaction (VAL643), while interaction with 4OQ3 was stronger (−7.71 kcal/mol; 2.19 µM), involving five alkyl bonds (ILE61, ILE99, LEU54, LEU57, VAL93), one hydrogen bond (GLY58), and one π–sigma bond (HIS96). Calciferol exhibited the strongest interaction for both proteins, with a binding energy of −10.13 kcal/mol (1T46; 1.27 µM), stabilized by twelve hydrophobic interactions (ALA621, VAL603, LYS623, CYS809, LEU644, CYS673, TYR672, LEU595, PHE811, LEU799, VAL654, VAL668). Against 4OQ3 (−10.28 kcal/mol; 9.5 nM), it formed five alkyl/π-alkyl bonds (VAL93, ILE99, HIS96, LEU54, TYR100) and one hydrogen bond (GLN72) (Figure 4). Communesin A showed moderate affinity to 1T46 (−4.73 kcal/mol; 283.01 µM), forming four carbon–hydrogen bonds (ILE789, ALA636, ASP810, GLU640) and three π-alkyl bonds (VAL643, LEU644, ARG791). For 4OQ3 (−8.32 kcal/mol; 778.66 nM), it established eight alkyl/π-alkyl interactions (VAL93, ILE61, PHE91, PHE86, LEU57, LEU82, ILE99, LEU54) and one π–sigma bond (HIS96). Cortisol bound strongly to both targets, with a docking score of −7.13 kcal/mol (1T46; 1.91 µM), forming three hydrogen bonds (ILE808, HIS790, GLU640) and two alkyl interactions (LEU783, VAL643). For 4OQ3 (−8.03 kcal/mol; 1.11 µM), it formed two hydrogen bonds (TYR100, GLN24) and four alkyl contacts (ILE19, LEU54, ILE99, HIS96). Myricetin demonstrated strong binding to 1T46 (−7.37 kcal/mol; 2.98 µM), with five hydrogen bonds (CYS673, GLU671, THR670, LYS623, GLU640), four π-alkyl contacts (ALA621, VAL603, VAL654, LEU644), one π–sulfur interaction (CYS809), and one π–sigma interaction (LEU799). Against 4OQ3 (−5.20 kcal/mol; 112.67 µM), it formed three hydrogen bonds (LEU54, MET62, GLN72), three π-alkyl bonds (ILE99, LEU57, VAL93), and one π–sigma bond (ILE61) (Supplementary Figure S3).

Comparing the two targets, both 5-epi-ilimaquinone and Andrastine D exhibited a clear binding preference for 4OQ3 over 1T46. 5-epi-ilimaquinone bound more tightly to 4OQ3 (−7.53 kcal/mol vs. −6.25 kcal/mol in 1T46) due to additional alkyl contacts with ILE99, ILE61, and LEU54. Similarly, Andrastine D demonstrated a marked increase in binding potency toward 4OQ3 (−7.71 kcal/mol vs. −5.33 kcal/mol in 1T46), driven by an expanded interaction network involving a key H-bond (GLY58), a π-sigma contact (HIS96), and five alkyl bonds. These interaction profiles indicate that both metabolites bind more favorably and selectively within the active site of 4OQ3.

Overall, among all evaluated ligands, Calciferol exhibited the strongest binding affinity toward both 1T46 and 4OQ3 target proteins. For the 1T46 receptor, Calciferol achieved a binding energy of −10.13 kcal/mol (Ki = 1.27 µM), which was predominantly stabilized by twelve alky/π-alkyl interactions. Against the 4OQ3 target, Calciferol demonstrated an even higher binding potency with a binding energy of −10.28 kcal/mol (Ki = 9.5 nM), anchored by one key hydrogen bond with GLN72 and five alky/π-alkyl interactions (Figure 4). These findings highlight Calciferol as the most promising candidate with superior dual-target binding potential.

## 3. Discussion

Among malignant diseases, breast cancer was the most commonly diagnosed cancer in females, and TNBC is the most difficult to treat. Researchers are racing to find solutions by trying to find more selective and safe anticancer agents, including this study exploring marine resources. The sponge that was obtained from Tanjung Kusu, Lembeh strait, Bitung City (Figure 1a) was identified as *Dactylospongia elegans* through morphological, spicule and genomic analysis identification (Figure 1b,c). *Dactylospongia elegans* was extracted using the maceration method with methanol and separated using a liquid–liquid separation method with ethyl acetate and water residue. The ethyl acetate fraction of *Dactylospongia elegans* contained terpenoids, steroids, and alkaloids that were detected using TLC methods (Supplementary Figure S1). Then, the ethyl acetate fraction of *Dactylospongia elegans* was separated using Open Column Chromatography using silica, resulting in ten subfractions that were identified using LC-MS/MS as Communesin A, Andrastine D, Cortisol, Myricetin, 5-epi-illimaquinone and Calciferol (Figure 2 and Figure S2, Table 1). The ethyl acetate fraction and F4 subfraction of *Dactylospongia elegans* demonstrated moderate cytotoxicity against MDA-MB-231 cells with IC_50_ values of 15.72 and 41.76 µg/mL, respectively, while the IC_50_ against was cisplatin 30.99 µg/mL (Table 3). This was accompanied by strong toxicity in the BSLT, particularly in fraction F6, with an LC_50_ = 32.83 µg/mL (Table 2). The morphology of MDA-MB-231 cells that were untreated was showed as a control (Figure 3a), and those treated with the ethyl acetate fraction of *Dactylospongia elegans* (Figure 3b) but not the F4 subfraction (Figure 3c) showed changes to apoptosis and cell cycle arrest compared with those treated with cisplatin (Figure 3d). Furthermore, molecular docking highlighted Calciferol as the most promising compound, exhibiting the strongest binding affinity toward both tyrosine kinase (1T46) and p53–MDM2 (4OQ3) receptors, suggesting a potential mechanism for apoptosis induction (Figure 4 and Figure S3 and Table 4).

The taxonomic identification of the collected sponge was initially supported by morphological observation and molecular analysis, showing an irregular lobed form, dark brown coloration, and a fibrous skeleton with smooth diactine megascleres and the absence of microscleres. These features are consistent with diagnostic characteristics of the genus *Dactylospongia*, which typically exhibit fibrous frameworks reinforced by diactinal spicules (Figure 1c) [12,13]. Compared with related species, *Dactylospongia elegans* displays massive lobate forms with reddish-brown coloration and abundant diactine megascleres, while *Dactylospongia metachromia* from Polynesia shows variable pigmentation and denser spicule bundles [14]. Taken together, the morphological, spicular, and molecular evidence reinforce the assignment of the present specimen to *Dactylospongia elegans*, while also highlighting intra-genus variations in skeletal architecture and pigmentation. From the DNA barcoding analysis, the DNA chain of the sample matches to *Dactylospongia elegans* strain SCS1 (GenBank: KY970158.1) and *Dactylospongia elegans* (GenBank: MW150456.1 and KC869554.1). From these results, we can confidently state that our sample is *Dactylospongia elegans*.

The methanol extract of *Dactylospongia elegans* was then separated using liquid–liquid separation with a funnel. After the ethyl acetate fractions that have any toxic and cytotoxic activity were detected, the fractions were separated using column chromatography. The chemical separation conducted by bioassay guided us to find the chemical compounds that have toxic and cytotoxic activity. TLC revealed the presence of multiple secondary metabolite classes in the ethyl acetate fraction of *Dactylospongia elegans*, as confirmed through distinct staining results with specific reagents [15]. TLC screening revealed the presence of multiple secondary metabolite classes in the ethyl acetate fraction of *Dactylospongia elegans*, specifically confirming the presence of terpenoids, steroids, and alkaloids, while phenolics were not detected [15]. Positive results for terpenoids and steroids were indicated by purple-to-gray bands after *p*-anisaldehyde–sulfuric acid and Liebermann–Burchard visualization, aligning with the known isoprenoid-based biosynthetic profile of marine sponges [13]. Alkaloid content was detected as orange-brown spots with Dragendorff’s reagent, suggesting nitrogen-containing bioactives potentially similar to those found in related Thorectidae species [16]. Conversely, phenolics were absent (negative reaction with FeCl_3_, a result consistent with findings in other demosponges [17]).

Table 1 displayed the application of liquid chromatography-tandem mass spectrometry (LC-MS/MS) in this study, which enabled sensitive and specific detection of the target compounds, with each analyte characterized by unique retention time and mass-to-charge (*m*/*z*) values [7]. Notably, metabolites such as Communesin A, Andrastine D, Myricetin, and Calciferol was confirmed by a close match between experimental and reference masses, corroborating previous reports on LC-MS’s utility for structural elucidation and quantification of both primary and secondary metabolites in marine-derived samples [18]. Among the detected metabolites, the peak corresponding to *m*/*z* 359.2 at 14.2 min was annotated as 5-epi-ilimaquinone. This study expands previous reports by demonstrating the presence of 5-epi-illimaquinone in bioactive fractions of *Dactylospongia elegans* and linking its cytotoxic potential to triple-negative breast cancer (MDA-MB-231) cells [11]. Chemically, 5-epi-illimaquinone features a lipophilic sesquiterpene scaffold combined with an electrophilic, redox-active quinonoid core. The lipophilic moiety facilitates cellular uptake across cancer cell membranes, while the quinonoid ring undergoes intracellular redox cycling to generate elevated reactive oxygen species (ROS) and acts as a Michael acceptor to alkylate key cellular nucleophiles. This dual chemical mechanism induces severe oxidative stress, mitochondrial dysfunction, and apoptotic signaling, explaining its observed cytotoxicity in MDA-MB-231 breast cancer cells [19].

The BSLT served as a preliminary screening tool to evaluate the general cytotoxic potential of the *Dactylospongia elegans* fractions, revealing a wide spectrum of toxicity. Table 2 shows the BSLT results for the *Dactylospongia elegans* fractions. Notably, subfractions F6 and F4 exhibited the most potent activity (LC_50_ = 32.831 and 55.485 µg/mL, respectively), suggesting they may contain lead compounds with a strong cytotoxic potential and may have relevance for further pharmacological evaluation. The BSLT is widely regarded as a cost-effective and rapid in vivo model for early cytotoxicity prediction, and has been positively correlated with the antitumor and anticancer properties of natural products in numerous studies [20]. A low LC_50_ value in this assay, particularly below 100 µg/mL, is generally considered indicative of bioactive compounds capable of inducing apoptosis or disrupting cellular metabolism [21]. Comparable findings were reported in previous study of *Dactylospongia elegans*, where crude extracts demonstrated LC_50_ values below 100 µg/mL in the BSLT assay supporting the notion that members of this genus consistently produce bioactive metabolites [22]. The present study, however, revealed a lower LC_50_ value for subfraction F6, indicating stronger cytotoxic potential than previously documented. The variation in lethality observed across *Dactylospongia elegans* subfractions likely reflects differences in the composition and concentration of bioactive metabolites in each subfraction, consistent with the inherent chemical diversity found in marine sponge-derived subfractions [13]. Taken together, these findings justify the prioritization of the most active fractions, particularly subfraction F6, for further bioassay-guided isolation and structural elucidation.

The cytotoxic potential of the ethyl acetate fraction from *Dactylospongia elegans* against the TNBC cell line MDA-MB-231 was strong, with an IC_50_ value of 15.72 ± 3 µg/mL, while subfraction F4 is 41.76 ± 0.5 µg/mL (Table 3). According to the U.S. National Cancer Institute and Geran’s protocol, compounds with IC_50_ values less than 20 µg/mL are considered strongly cytotoxic [23]. In comparison, the F4 subfraction and the positive control cisplatin exhibited much lower IC_50_ values of 41.76 ± 0.5 and 30.99 ± 3 µg/mL (Table 3), indicating moderate cytotoxicity. From this result, the ethyl acetate fraction of *Dactylospongia elegans* is more cytotoxic than cisplatin. This highlights the need to observe the presence of bioactive secondary metabolites within *Dactylospongia elegans* that warrant further purification and characterization. This is in accordance with a previous study that reports the IC_50_ value of 5-epi-Ilimaquinone against 501Mel melanoma cells to be 7.88 µg/mL, which is also a potent cytotoxic effect [11]. However, the F4 subfraction of *Dactylospongia elegans* had an IC_50_ that showed moderate cytotoxicity; it may be that the F4 subfraction was not 5-epi-Ilimaquinone or there were synergistic effects between compounds that occurred in the ethyl acetate fraction of *Dactylospongia elegans*. Usually, natural product fractions often exhibit reduced potency compared with single purified drugs due to the complex mixture of compounds, yet they remain valuable as reservoirs of novel lead molecules with the potential to be optimized for anticancer therapy.

The morphological assessment of MDA-MB-231 cells following cytotoxic testing using the resazurin assay revealed notable differences between untreated and treated groups (Figure 3). In the untreated group (Figure 3a), cells exhibited typical fibroblast-like morphology, characterized by elongated spindle shapes, firm adherence, and near-confluent growth. In contrast, cells observed post-treatment with the ethyl acetate fraction of *Dactylospongia elegans* (Figure 3b) showed a reduced number of adherent cells, increased rounding, shrinkage (red arrows), and the presence of condensed bodies or blebbing (blue arrows), suggesting a loss of viability [24]. Moreover, MDA-MB-231 treated with the ethyl acetate fraction of *Dactylospongia elegans* had numbers that also seemed much less numerous than untreated group (Figure 3a) and the group treated with cisplatin (Figure 3d). Compared with the MDA-MB-231 cells that were treated with cisplatin (Figure 3d), apoptosis occurred in the same way but the numbers were approximately the same without any treatment. While MDA-MB-231 treated with the F4 subfraction of *Dactylospongia elegans* (Figure 3c) showed some apoptosis occurrence signs, they were not as intense as in those treated with cisplatin (Figure 3d). These changes are consistent with early features of apoptosis, although they are not definitive without further confirmatory testing. Despite the limitations of morphological analysis alone, these findings align with the cytotoxic effect detected in the resazurin assay, indicating that the test fraction likely compromises cell viability through a mechanism suggestive of apoptosis and cell cycle arrest [25].

The RMSD is commonly applied to measure the positional difference between predicted and reference ligand conformations. An RMSD below 2 Å is generally considered indicative of a reliable binding mode prediction [26]. Table 5 presents the molecular interaction profiles of six ligands derived from *Dactylospongia elegans*, evaluated through in silico analysis against the tyrosine kinase receptor (PDB ID: 1T46) and the p53–MDM2 complex (PDB ID: 4OQ3) in MDA-MB-231 breast cancer cells. The 1T46 receptor was selected because tyrosine kinase is known to be overexpressed in a subset of TNBC and plays a role in promoting cell migration and invasiveness in MDA-MB-231 cells, making it a relevant therapeutic target [27]. In addition, the p53–MDM2 complex was chosen because MDM2 is a protein that suppresses the function of the tumor suppressor p53. Inhibiting MDM2 has shown promising therapeutic potential in TNBC models. Restoring p53 activity through MDM2 inhibition can trigger apoptosis in cancer cells, as p53 regulates the expression of pro-apoptotic genes such as Bax and PUMA, thereby promoting programmed cell death in tumor cells [28].

A re-docking process of the native ligand with each receptor was performed, and succeeded with RMSD values < 2Å, to evaluate the accuracy of docking. Binding energy reflects the thermodynamic favorability of the ligand–receptor interaction, where more negative values indicate stronger and more spontaneous binding, consistent with Gibbs free energy (ΔG) principles [29]. In molecular docking studies, a binding energy below −5 kcal/mol is widely considered indicative of significant ligand–receptor interaction [30]. In parallel, the inhibition constant (Ki) serves as a complementary indicator of ligand potency. Lower Ki values correspond to a higher affinity, meaning a smaller amount of the compound is required to inhibit receptor activity, which strengthens its potential as a drug candidate [31]. Binding energies ranged from −4.73 kcal/mol to −10.13 kcal/mol for 1T46 and from −5.20 kcal/mol to −10.28 kcal/mol for 4OQ3, indicating stronger overall binding to the latter. For the tyrosine kinase receptor (1T46), the majority of ligands exhibited moderate to strong inhibitory activity, with Ki values ranging from 1.27 μM to 283.01 μM. For the p53–MDM2 complex (4OQ3), overall stronger inhibition was observed, with several ligands achieving sub-micromolar to micromolar Ki values (9.5 nM–112.67 μM). In terms of binding interactions, the 4OQ3 complex exhibited a more extensive and diverse network including multiple alkyl, π-alkyl, and hydrogen bonds compared to 1T46, indicating a more favorable binding landscape for the tested ligands. This may be attributed to the structural conformation of the p53–MDM2 binding pocket, which provides more accessible hydrophobic regions and polar residues that facilitate stable ligand accommodation [32]. Calciferol, a vitamin D derivative of marine origin, exhibited the strongest binding affinity among all tested ligands, with binding energies of −10.13 kcal/mol against 1T46 and 10.28 kcal/mol against 4OQ3, alongside notably low inhibition constants of 1.27 µM and 9.5 nM, respectively. These results are in line with previous reports demonstrating that vitamin D analogs can exert antiproliferative and pro-apoptotic effects in triple-negative breast cancer models, including MDA-MB-231 cells [33]. Overall, the docking results suggest that the compounds from *Dactylospongia elegans* possess the potential to exert antiproliferative effects through interaction with tyrosine kinase and p53–MDM2 targets, supporting their prospective role in apoptosis regulation in MDA-MB-231 cells.

Cultivation of the sponge *Dactylospongia elegans* is ongoing. Further research should include isolation of active compounds and detailed characterization of active compounds, detailed investigation of molecular pathways such as apoptosis and cell cycle regulation and toxicity assessment against normal cell lines to ensure therapeutic selectivity, along with in vivo validation of efficacy and safety.

## 4. Materials and Methods

### 4.1. Materials

The materials used in this study included the breast cancer cell line MDA-MB-231, obtained from the European Collection of Authenticated Cell Cultures (ECACC, Salisbury, UK). Analytical instruments and laboratory equipment included a rotary evaporator (BÜCHI Labortechnik AG, Flawil, Switzerland), freeze dryer LGJ-10 (AELAB GUANGZHOU Co., Ltd., Guangzhou, China), Infinite M200 PRO microplate reader (Tecan Group Ltd., Männedorf, Switzerland), biosafety cabinet class II type B2 Esco Micro Pte. Ltd. (Changi, Singapore), and standard laboratory glassware and plasticware used throughout the extraction, chromatography, and cytotoxicity assay procedures.

### 4.2. Biological Material

The sponge was collected by scuba diving at depths of 7–15 m from dive point Tanjung Kusu Kusu, Lembeh Strait, Bitung City, North Sulawesi Province, Indonesia with coordinates 01°27.240′ N; 125°14.371′ E (Figure 1a) in September 2023. Prior to the extraction, the sample was promptly frozen and stored in the National Research and Innovation Agency’s (BRIN) Genomics Laboratory in Cibinong, West Java, Indonesia. Through morphological study and spicule analysis, the specimen was recognized as belonging to the *Dactylospongia* sp. (Figure 1c).

### 4.3. Sponge Genomic Identification

DNA from the sponge was extracted using Cetyltrimethylammonium Bromide Extraction Method, which was amplified with primer NL1-NL2 using KOD FX Neo kit (Toyobo, Osaka, Japan). DNA visualized in Agarose Gel, and 2-way sequenced using Sanger DNA Sequencing by using Capillary Electrophoresis by 1st BASE, Axil Scientific Pte Ltd. (JTC MedTech Hub, Singapore). The DNA chain that obtained matches with the database in NCBI for species identification.

### 4.4. Extraction and Fractionation of Sponge Dactylospongia elegans

The extraction process was conducted using the maceration method. The sponge (350 g) was cut into small pieces and soaked in methanol (MeOH) overnight. The filtrate was collected, and the residue was subjected to methanol. The process was repeated for the residue–methanol mixture until the third day. After maceration, a Rotary Vacuum Evaporator (Rotavapor Buchi R-300, BÜCHI Labortechnik AG, Flawil, Switzerland) was used to mix and condense all of the filtrate. The residue was then thoroughly separated using ethyl acetate (EtOAc) and water.

### 4.5. Identification of Secondary Metabolites

The identification was done using the TLC method. The fraction was spotted on a silica gel 60 F254 (Merck KGaA, Darmstadt, Germany). The optimization of eluent was carried out to determine the best fluid elution profile. The eluent for terpenoid was hexane:ethyl acetate (4:1) with an Anisaldehyde Sulfate stain, heating at 105 °C for 5–10 min [15,34]. The alkaloid eluent was hexane:ethyl acetate (4:1) with a Dragendorff stain. Steroid eluent was hexane:ethyl acetate (3:2) with a Lieberman Bouchardat stain, heating at 105 °C for 5–10 min. The phenolic eluent was hexane:ethyl acetate (4:1) with FeCl_3_ staining. Universal compounds were also identified using H_2_SO_4_ where the eluent was hexane: ethyl acetate (4:1) [15].

### 4.6. Open Column Chromatography by Silica

Open column chromatography with an ethyl acetate–hexane–methanol gradient system was used to separate the ethyl acetate fractions. Methods including trapping the fractions in each band, eliminating the solvent, and enabling the anti-cancer test were applied.

### 4.7. LC-MS Analysis

LC–MS analyses were performed on an Agilent Revident LC/Q-TOF system equipped with a ZORBAX Eclipse Plus C18 RRHD column (2.1 mm × 100 mm, 1.8 μm) from Agilent Technologies (Santa Clara, CA, USA). Separation was achieved using a gradient elution with formic acid 0.1% in water (eluent A) and acetonitrile with formic acid 0.1% (eluent B) at a flow rate of 0.3 mL/min. The injection volume was 10 μL, and the total run time was 19 min. LC-MS analysis was conducted to characterize the chemical constituents in the ethyl acetate fraction of the sponge. This technique integrates liquid chromatography, which separates compounds based on polarity and retention time, with mass spectrometry for detecting molecular ions and determining molecular weights. The resulting mass-to-charge (*m*/*z*) data facilitated the identification of metabolites by comparing them to known or predicted molecular formulas, supporting the profiling of potential bioactive compounds.

### 4.8. Toxicity Assay Using Brine Shrimp Lethality Test (BSLT)

This study used a modified BSLT to assess the toxicity of a test solution. Brine shrimp larvae (Artemia Supreme Plus, Great Salt Lake, UT, USA) were hatched in a standardized artificial seawater environment over 48 h, using a controlled light source and aeration. To conduct the test, the solution was diluted to eight different concentrations, ranging from 7.8125 to 1000 micrograms per milliliter (µg/mL). Ten brine shrimp larvae were then placed into individual wells of a 96-well plate, with each concentration being tested in triplicate (three wells per concentration). The larvae were exposed to the solutions for 24 h at room temperature, under a controlled light. Following the exposure period, the number of surviving larvae in each well was counted. To establish a baseline, a negative control group was included, where larvae were exposed to artificial seawater without the test solution. Finally, the median lethal concentration (LC_50_), which represents the concentration of the test solution that killed 50% of the larvae, was calculated using probit analysis.

### 4.9. Cytotoxic Examination Against MDA-MB-231 Cells

The cytotoxicity of the compounds was assessed using the TOX-8 assay method. MDA-MB-231 cells obtained from ECACC were grown in Dulbecco’s Minimum Essential Medium/DMEM (Sigma Aldrich; Merck KGaA, Darmstadt, Germany) liquid culture medium containing 10% fetal bovine serum (FBS) (Gibco, Grand Island, NY, USA) and 1% penicillin–streptomycin (Gibco). Trypsin-EDTA (Gibco), Phosphate Buffered Saline (PBS, Sigma Aldrich), and TOX-8 reagent kit (Sigma-Aldrich). Dimethyl sulfoxide (DMSO, Sigma Aldrich) was used as a solvent and as negative control. In addition, the cells were distributed at a density of 10,000 cells/well into 96-well plates and incubated for 24 h at 37 °C and 5% CO_2_ gas. Treatment of ethyl acetate fraction of *Dactylospongia elegans* was then conducted with eight concentration series of samples, namely 1000 µg/mL, 500 µg/mL, 250 µg/mL, 125 µg/mL, 62.5 µg/mL, 31.25 µg/mL, 15.625 µg/mL, and 7.8125 µg/mL for 24 h. The F4 fractions of *Dactylospongia elegans* are 45 µg/mL, 22.5 µg/mL, 11.25 µg/mL, 5.625 µg/mL, 2.8125 µg/mL, 1.40625 µg/mL, 0.703125 µg/mL, and 0.3515625 µg/mL. In this context, the concentration of the compounds was tested in three parallel experiments. Cisplatin was used as a positive control in this test. The wells containing only culture media served as media controls, while those without a compound served as cell, DMSO, and positive controls. Cell morphology was observed using inverted microscope Axio Vert.A1 (Zeiss, Oberkochen, Baden-Württemberg, Germany). After overnight incubation, 100 µL of TOX-8 reagent (Thermo Fisher Scientific, Waltham, MA, USA) was added to each well and re-incubated for 1 h until a color change was reported. The intensity of color changes was read using a microplate reader Infinite M200 PRO multimode (Tecan, Männedorf, Switzerland) at 570 nm and 600 nm (reference wavelength). From the measurement results, the percentage of cell growth inhibition was calculated using the equation [25].

% Cell Inhibition = 100 − ((Absorbance of test − Absorbance of blank)/(Absorbance of control − Absorbance of blank)) × 100%

IC_50_ values were calculated by Logarithmic regression method using Microsoft Excel software.

### 4.10. Molecular Docking

Molecular docking was conducted to examine how the compounds interact within the active site of tyrosine kinase receptors. The 3D crystal structure of the receptor (PDB ID: 1T46) was retrieved from the PDB. Prior to docking, the protein structures were prepared by removing water molecules and native ligands using BIOVIA Discovery Studio (2026‌). Native ligands were re-docked to validate the docking protocol, achieving RMSD values below 2 Å. Compound structures were energy-minimized using Chem3D Pro 12.0 (PerkinElmer, Inc., Shelton, CT, USA), then docked into the receptor’s active site using AutoDockTools 1.5.6 (CCSB, San Diego, CA, USA) with a grid box size of 40 × 40 × 40. The resulting ligand–receptor complexes were analyzed using BIOVIA Discovery Studio.

## 5. Conclusions

In conclusion, this study evaluated the cytotoxic and toxic potential of the ethyl acetate fraction of *Dactylospongia elegans* against the triple-negative breast cancer cell line MDA-MB-231. LC-MS/MS analysis identified several predicted metabolites including 5-epi-ilimaquinone, a sesquiterpene quinone and Calciferol, known for its anticancer potential. This is further supported by molecular docking results. These findings suggest that the active constituents in *Dactylospongia elegans* may exert antiproliferative effects through interaction with key cancer-related targets. Overall, this marine-derived fraction presents promising potential as a source of lead compounds for triple-negative breast cancer therapy.

## Figures and Tables

**Table 1:** LC-MS data of *Dactyospongia elegans* fraction.

Retention Time (Minute)	Fragment (*m*/*z*)	Reference Fragment (*m*/*z*)	Mass Calculation (*m*/*z*)	Reference Mass Calculation (*m*/*z*)	Predicted Compound (Group)	Source	Citation (Prefix MassBank ID: MSBNK-)
9. 559	194.1174 385.2007 314.3417 325.2348 343.1903 384.2007 457.2191	194.0964 385.2011 314.1667 325.1444 343.1936 385.2027 457.2602	456.2125	456.25250	Communesin A (Alkaloid)	Marine algae *Enteromorpha* *intestinalis*	Massbank (HBM4EU-HB003902)
11. 692	151.039 341.2481 355.2277 429.3186	151.0966 341.2484 355.2818 429.263	428.3112	428.25629	Andrastine D (Meroterpenoid)	Marine derived fungus *Penicillium* sp.	Mass bank (AAFC-AC000821)
11. 741	151.0965 297.2401 298.2436 363.3108	151.0767 297.1837 298.1829 363.2166	362.2028	362.2093	Cortisol (Steroid)	Marine hormone	Massbank (Antwerp_Univ-METOX_N105306_EF88)
11. 776	133.0790 151.0965 289.1541 290.1576 318.2775	133.0654 151.0018 289.024 290.0244 318.2370	319.2848	319.25	Myricetin (Flavonoid)	Marine-derived polyphenols	Massbank (Fiocruz-FIO00188)
12. 341	355.2815 356.2855 357.2879	-	354.2745	358.5	5-epi-ilimaquinone (Sesquiterpene quinone)	*Dactylospongia elegans*	[11]
14. 573	98.9843 111.0919 121.0509 341.2654 355.2808 397.3275	98.5079 111.020 121.0656 341.002 356.0340 397.347	396.3203	396.339	Calciferol (Fat)	Marine algae	Massbank (RIKEN-PR100476

**Table 2:** BSLT test results for the *Dactylospongia elegans* subfraction.

Subfraction	LC_50_ (µg/mL)	Classification
F1	658.118	Low
F2	307.407	Moderate
F3	67.302	High
F4	55.485	High
F5	60.141	High
F6	32.831	High
F7	44.194	High
F8	1174.19	Not Toxic
F9	195.396	Moderate
F10	1000	Low

**Table 3:** Inhibition concentration of 50% cell death (IC_50_) value against MDA-MB-231.

No	Sample	IC_50_ (µg/mL)
1	Cisplatin	30.99 ± 3
2	EtOAc fraction *Dactylospongia elegans*	15.72 ± 3
3	F4 subfraction *Dactylospongia elegans*	41.76 ± 0.5

**Table 4:** Validation of molecular docking using native ligand.

Compound	Receptor	Reference RMSD	Binding Energy (kcal/mol)	Ki	Bond
4- 4-methyl-piperazin-1-yl methyl [4-methyl-3-(4-pyridin-3-yl-pyrimidin-2-ylamino)-phenyl]-benzamide (Natural Ligand)	1T46 (Tyrosine Kinase)	0.88	−13.53	120.18 (pM)	VAL A:603; VAL A:622; VAL A:668; LEU A:595; LEU A:799; LEU A:783; LEU A:644; HIS A:790; THR A:670; ALA A:621; PHE A:811; LYS A:623; ASP A:810; CYS A:673; GLU A:640; VAL A:654;
1-(5-chloro-2-methylphenyl)-5-(3-chlorophenyl)-2-(3-methylphenyl)-1H-imidazole-4-carboxylic acid	4OQ3 (p53-MDM2)	0.49	−10.96	9.22 nM	PHE C:86, TYR C:67, PHE C:91, ILE C:61, VAL C:93, LYS C:94, HIS C:96, TYR C:100, LEU C:54, ILE C:99

**Table 5:** Molecular docking results of predicted active compounds contained in ethyl acetate fraction of *Dactylospongia elegans*.

Compound	Receptor	Cluster	Binding Energy (kcal/mol)	Ki (μM)	Bond
5-epi-illimaquinone	1T46 (Tyrosine Kinase)	39	−6.25	26.07 μM	GLU A:640, VAL A:643, HIS:790
	4OQ3 (p53-MDM2)	79	−7.53	2.9 μM	VAL C:93, LEU C:54, ILE C:61, ILE C:99
Andrastine D	1T46 (Tyrosine Kinase)	91	−5.33	67.88 μM	VAL A:643
	4OQ3 (p53-MDM2)	83	−7.71	2.19 μM	VAL C:93, GLY C:58, ILE C:61, ILE C:99, LEU C:54, LEU C:57, HIS C:96
Calciferol	1T46 (Tyrosine Kinase)	64	−10.13	1.27 μM	ALA A:621, VAL A:603, LYS A:623, LEU A:644, CYS A:809, VAL A:668, VAL A:654, PHE A:811, LEU A:799, LEU A:595, TYR A:672, CYS A:673
	4OQ3 (p53-MDM2)	87	−10.28	9.5 nM	GLN C:72, VAL C:93, ILE C:99, HIS C:96, LEU C:54, TYR C:100
Communesin A	1T46 (Tyrosine Kinase)	100	−4.73	283.01 μM	ARG A:791, ILE A:789, VAL A:643, LEU A:644, GLU A:640, ASP A:810, ALA A:636
	4OQ3 (p53-MDM2)	100	−8.32	778.66 nM	VAL C:93, HIS C:96, LEU C:54, ILE C:99, LEU C:57, LEU C:82, PHE C:86, PHE C:91, ILE C:61
Cortisol	1T46 (Tyrosine Kinase)	8	−7.13	1.91 μM	ILE A:808, HIS A:790, GLU A:640, LEU A:783, VAL A:643
	4OQ3 (p53-MDM2)	8	−8.03	1.11 μM	LEU C:54, ILE C:99, HIS C:96, TYR C:100, ILE C:19, GLN C:24
Myricetin	1T46 (Tyrosine Kinase)	77	−7.37	2.98 μM	LEU A:799, CYS A:673, GLU A:671, ALA A:621, VAL A:603, THR A:670, VAL A:654, LEU A:644, CYS A:809, LYS A:623, GLU A:640
	4OQ3 (p53-MDM2)	65	−5.20	112.67 μM	LEU C:54, MET C:62, GLN C:72, ILE C:61, VAL C:93, LEU C:57, ILE C:99

## Data Availability

The data supporting the findings of this study are available from the corresponding author upon reasonable request.

## Supplementary Materials

The following supporting information can be downloaded at: https://www.mdpi.com/article/10.3390/md24080271/s1, Figure S1: Thin layer chromatography pattern of *Dactylospongia elegans* ethyl acetate fraction’s compound group determination; Figure S2: LC-MS/MS results of the secondary metabolites from the ethyl acetate fraction of *Dactylospongia elegans* except for active compounds that are suspected to be responsible for cytotoxic activity; Figure S3: Ligand–receptor interaction of *Dactylospongia elegans* secondary metabolite compounds except for active compounds that are suspected to be responsible for cytotoxic activity.

## Abbreviations

The following abbreviations are used in this manuscript:

MDA-MB	M.D. Anderson-Metastasis Breast
TNBC	Triple-Negative Breast Cancer
BSLT	Brine Shrimp Lethality Test
p53-MDM2	Tumor Protein 53-Mouse Double Minute 2
TLC	Thin Layer Chromatography
LC-MS	Liquid Chromatography-Mass Spectrophotometry
EtOAc	Ethyl Acetate
IC	Inhibitory Concentration
RMSD	Root Mean Square Deviation
Ki	Inhibitor Constant
PDB	Protein Data Bank
