# Supplementary material for: Cytotoxic Activity and In Silico Study of Secondary Metabolites Derived from Dactylospongia elegans
Source: Mar Drugs. 2026 Aug 4;24(8):271. doi: 10.3390/md24080271 (PMC13514943; doi:10.3390/md24080271)
Supplement: Supplementary file 1 [file marinedrugs-24-00271-s001.zip › marinedrugs-4465917-supplementary.pdf]

Supporting Information

Cytotoxic activity and in silico Study of Secondary Metabolites derived from Dactylospongia elegans

Yuni Elsa Hadisaputri[1,2*], Nafisa Nurfatia Hidayat[1], Tutik Murniasih[3], Ariyono Hadi3, Mutakin Mutakin[4], Elin Julianti[5*], Nunung Yuniati[6], Yonathan Asikin[7,8]

* **Correspondence:** yuni.elsa@unpad.ac.id; elin_julianti@itb.ac.id

List of Supporting Information

Figure S1. Thin Layer Chromatography pattern of *Dactylospongia elegans* ethyl acetate 2
Figure S2. LC-MS/MS results of the secondary metabolites from the ethyl acetate fraction of *Dactylospongia elegans* except for active compounds that are suspected to be responsible for cytotoxic activity 3
Figure S3. Ligand-receptors interaction of Dactylospongia elegans secondary metabolites compounds except for active compounds that are suspected to be responsible for cytotoxic activity 4

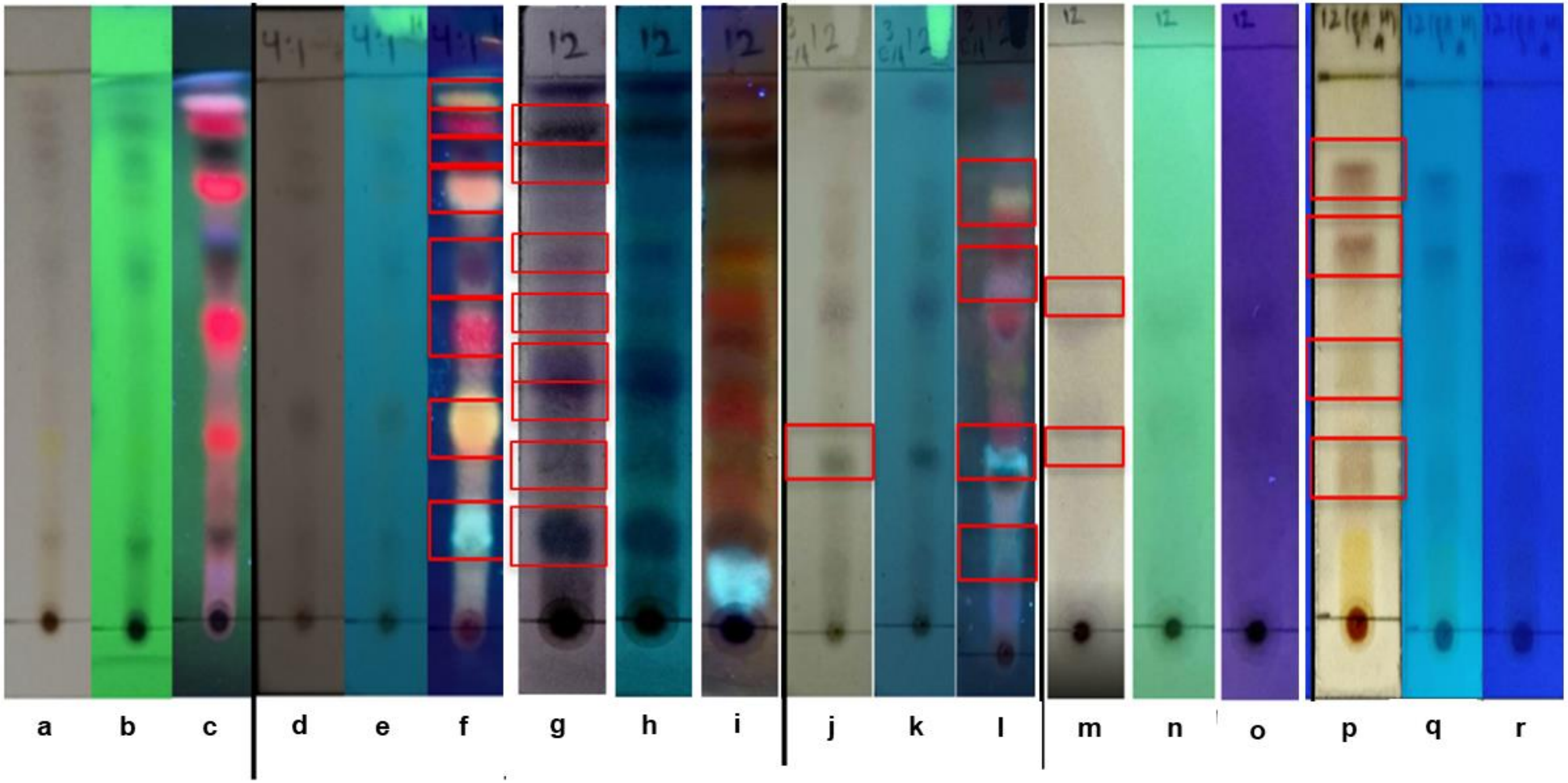

Figure S1. Supplementary Figure 1. Thin Layer Chromatography pattern of *Dactylospongia elegans* ethyl acetate fraction's compounds group determination, with solvent n-hexane:ethyl acetate (4:1), (a) before sprayed with any reagent, (b) under UV254 light and (c) UV366 light. (d) Visible after being sprayed with H_2SO_4 5%, (e) under UV254 light and (f) UV366 light. While (g) visible after sprayed with Anisaldehyde solution reagent, (h) under UV254 light and (i) UV366 light, (j) visible after sprayed with Lieberman–Burchard reagent, (k) under UV254 light and (l) UV366 light. Then (m) visible after sprayed with $FeCl_3$ reagent, (n) under UV254 light and (o) UV366 light. Moreover, (p) visible after sprayed with dragendorff reagent, (q) under UV254 light and (r) UV366 light. Spot detected in every plate which sprayed each reagent (red box).

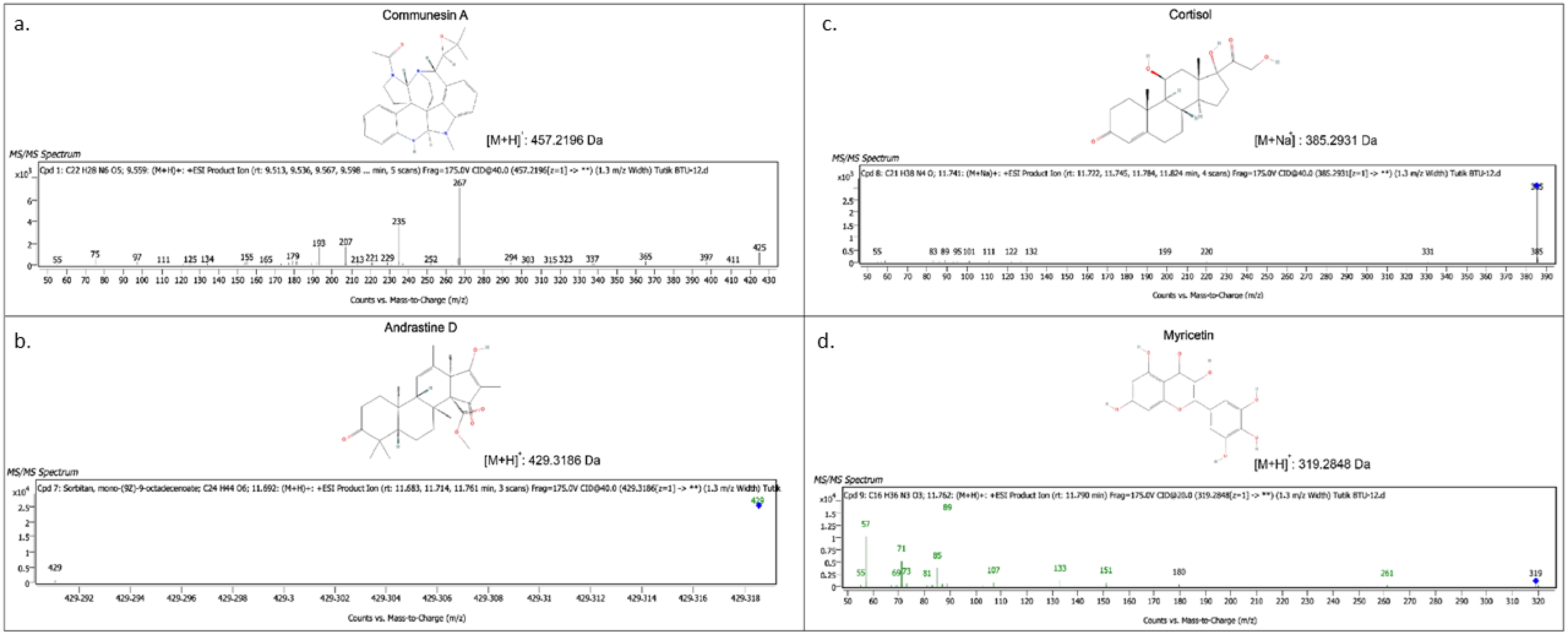

Figure S2. LC-MS/MS results of the secondary metabolites from the ethyl acetate fraction of *Dactylospongia elegans* except for active compounds that are suspected to be responsible for cytotoxic activity. (a) Structure and MS spectrum of Communesin A, (b) Andrastine D, and (c) Cortisol and (d) Myricetin.

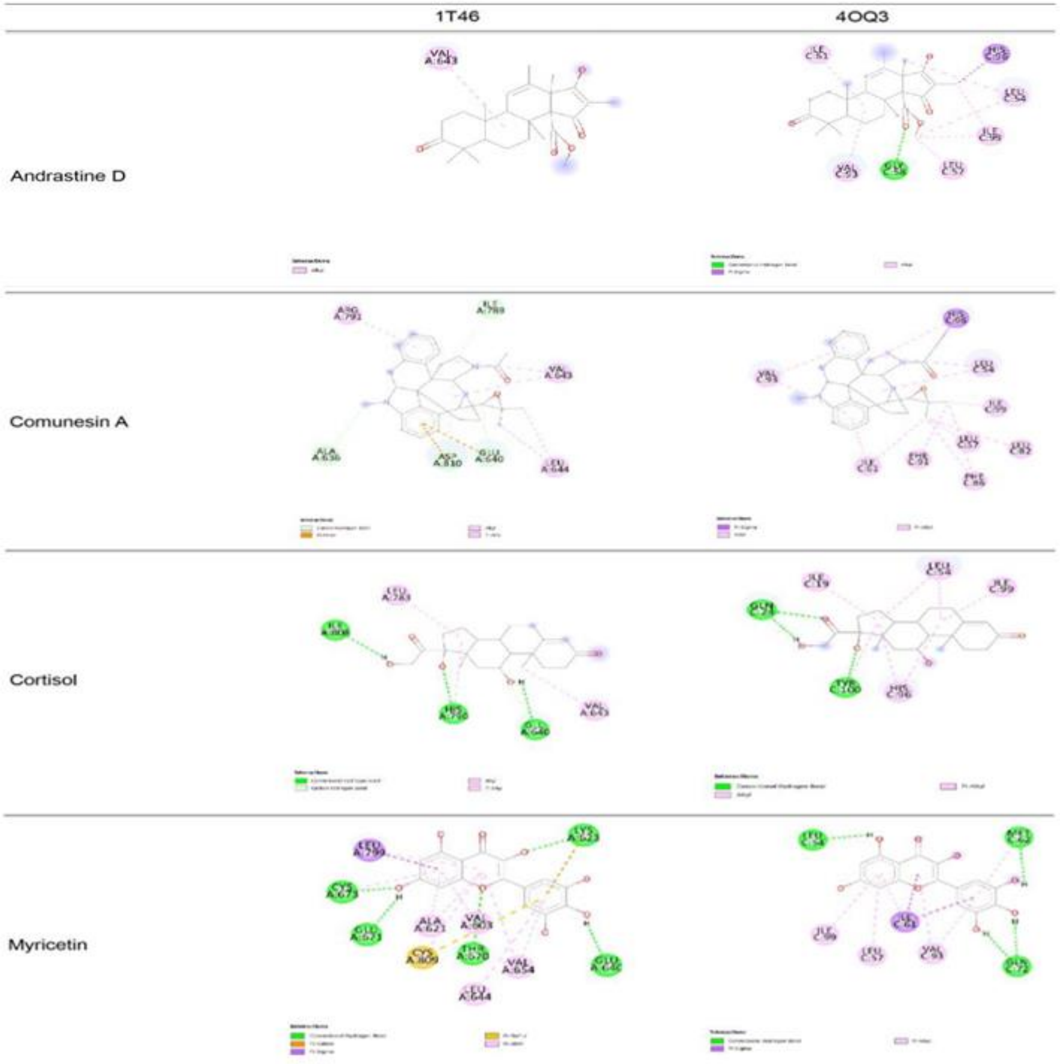

Figure S3. Ligand-receptors interaction of *Dactylospongia elegans* secondary metabolites compounds except for active compounds that are suspected to be responsible for cytotoxic activity. Ligand-receptors interaction of Communesin A, Andrastine D, and Cortisol and Myricetin.
